# Radiotherapy for overcoming immune checkpoint inhibitor resistance in esophageal squamous cell carcinoma

**DOI:** 10.3389/fimmu.2026.1905086

**Published:** 2026-09-14

**Authors:** Changjiang Du, Yuanfei Li

**Affiliations:** 1 First Clinical Medical College of Shanxi Medical University, Taiyuan, Shanxi, China; 2 Department of Oncology Digestive, First Hospital of Shanxi Medical University, Taiyuan, Shanxi, China

**Keywords:** cancer-associated fibroblasts, esophageal squamous cell carcinoma, immune checkpoint inhibitor, immunotherapy resistance, radiotherapy, tumor microenvironment

## Abstract

Immunotherapy has improved outcomes for patients with esophageal squamous cell carcinoma (ESCC), yet a substantial proportion develop resistance. The tumor microenvironment (TME) drives this resistance through four hallmarks: physical barriers formed by cancer-associated fibroblast (CAF)-deposited extracellular matrix (ECM) and elevated interstitial fluid pressure (IFP), enrichment of immunosuppressive cells, effector T cell exhaustion, and impaired antigen presentation. Radiotherapy, a cornerstone of ESCC treatment, has the potential to simultaneously target these barriers through multiple complementary mechanisms. Preclinical evidence suggests that high-dose or ablative radiotherapy can drive CAFs into irreversible senescence, triggering a senescence-associated secretory phenotype (SASP) that upregulates matrix metalloproteinases (MMPs) and degrades ECM. In preclinical models, IFP can be reduced through vascular normalization (low-dose) or tumor debulking (high-dose). There is also evidence, largely from experimental systems, that radiotherapy may eliminate immunosuppressive cells, ameliorate T cell exhaustion, and upregulate MHC class I and PD-L1 expression to restore immune recognition. These immunomodulatory effects appear to be dose- and fractionation-dependent; conventional fractionation often falls below the threshold required to dismantle the physical barrier—a factor that is hypothesized to contribute to, but does not fully account for, the limited success of chemo-immuno-radiotherapy trials to date. Building on this mechanistic framework, we evaluate clinical evidence for radiotherapy-immunotherapy combinations in ICI-resistant ESCC, compare the distinct roles of stereotactic body radiotherapy (SBRT) and low-dose radiotherapy (LDRT), and propose a “barrier-exhaustion score” as a hypothetical tool to guide individualized treatment. This review provides a mechanistic rationale and clinical perspective for radiotherapy-based strategies to overcome immunotherapy resistance in ESCC.

## 1 Introduction

Esophageal cancer is among the most prevalent malignancies worldwide. According to GLOBOCAN 2022 data, an estimated 511,000 new cases and 445,000 deaths occurred in 2022, making it the 11th most commonly diagnosed cancer and the 7th leading cause of cancer death globally (1).The two major histological subtypes—esophageal squamous cell carcinoma (ESCC) and esophageal adenocarcinoma (EAC)—exhibit strikingly different geographical distributions. ESCC predominates in East Asia, Eastern Africa, and parts of South America, where it is strongly linked to tobacco and alcohol use, whereas EAC is more common in Western Europe and North America, where obesity and gastroesophageal reflux are major risk factors. In China, which bears the highest absolute burden of esophageal cancer worldwide, over 90% of cases are ESCC (1). In recent years, phase III clinical trials such as CheckMate-648 and KEYNOTE-590 have established immunotherapy as a cornerstone in advanced esophageal cancer (2, 3). Studies such as ESCORT-NEO/NCCES01 have extended its application to the neoadjuvant and adjuvant settings for locally advanced disease (4). Emerging evidence suggests that the benefit of immunotherapy may differ between subtypes: in the CheckMate-577 trial, the disease-free survival advantage conferred by adjuvant nivolumab was more pronounced in patients with squamous cell carcinoma than in those with adenocarcinoma (5). Given the preponderance of ESCC in regions where immunotherapy is most intensively studied, and the particular challenge of resistance in this subtype, the present review focuses on ESCC. Despite these advances, acquired resistance remains a pervasive clinical challenge. In the CheckMate-648 and KEYNOTE-590 trials, only 45–53% of patients with advanced ESCC achieved an objective response to first-line immunochemotherapy, and the median duration of response among responders was limited to 7–11 months (2, 3). A recent multicenter study further reported that among ESCC patients who initially benefited from first-line ICI-based therapy, the majority eventually developed disease progression, with nearly all events occurring within two years (6). These data underscore that resistance is an almost inevitable consequence of current immunotherapy, highlighting the urgent need for strategies to overcome it. For patients who develop resistance, therapeutic options remain limited and unsatisfactory. Single-agent chemotherapy(docetaxel, nab-paclitaxel, oririnotecan) have shown only a median survival of 3 months in ESCC (7); the combination of ICI rechallenge with anti-angiogenic therapy has yielded a median PFS of up to 6 months (8, 9); and the long-term efficacy of bispecific antibodies in the post-immunotherapy setting remains to be determined.

Radiotherapy, a mainstay of esophageal cancer treatment, has emerged as a promising partner for immunotherapy. The abscopal effect of radiotherapy and its systemic immunomodulatory roles have been increasingly recognized. Radiotherapy not only provides local disease control but can also reshape the tumor microenvironment into an immunostimulatory state, potentially reversing ICI resistance and creating a window for re-challenge with immune checkpoint inhibitors.

Although radiotherapy has been recognized as a promising partner for immunotherapy, the mechanistic basis for how it can specifically reverse established ICI resistance in ESCC—and how radiotherapy parameters should be optimized for this purpose—has not been systematically examined. The review provides an in-depth discussion of the processes through which radiotherapy reverses immunotherapy resistance in ESCC. We first outline the four microenvironmental hallmarks of resistance, namely physical barriers, enrichment of immunosuppressive cells, effector cell dysfunction and impaired antigen presentation and then systematically discuss how radiotherapy can overcome each of these barriers. Lastly, we explain how these mechanistic understandings can guide rational choices regarding the modalities of radiotherapy (SBRT vs. LDRT) and individualized post-resistance therapeutic strategies.

Throughout this review, we distinguish three levels of evidence. Level 1 refers to data obtained directly from human ESCC specimens or esophageal cancer cell lines. Level 2 refers to clinical evidence from other cancer types. Level 3 refers to preclinical data from animal models or *in vitro* systems, which should be regarded as hypothesis-generating. Where a mechanism is inferred from non-esophageal models, this is explicitly stated. Unless otherwise specified, all statements refer to Level 1 evidence.

## 2 Microenvironmental characteristics of immunotherapy resistance in esophageal cancer

The complexity of the tumor microenvironment (TME) in esophageal squamous cell carcinoma (ESCC) is the root cause of immunotherapy resistance. The features that drive this resistance can be summarized into four major aspects: physical barriers, enrichment of immunosuppressive cells, effector cell dysfunction, and impaired antigen presentation.

### 2.1 Physical barrier and immune exclusion

In the tumor microenvironment (TME) of most solid tumors, activated cancer-associated fibroblasts (CAFs) synthesize and deposit collagen fibers, forming a rich extracellular matrix (ECM). Simultaneously, they secrete lysyl oxidase (*LOX*) to promote collagen cross-linking, thereby further increasing ECM stiffness and establishing a dense physical barrier (10, 11). In ESCC, this barrier function is executed with remarkable spatial precision. A distinct subpopulation of CAFs characterized by *COL11A1* expression (*COL11A1*+ CAFs) has been identified as the dominant fibrosis-driving subset at the tumor–stroma interface. These cells exhibit pronounced activation of extracellular matrix remodeling programs, including upregulated expression of structural collagens and collagen-modifying enzymes such as lysyl oxidase-like 2 (*LOXL2*), *LOX*, and pro-fibrotic factors of the *TGF-β* family (12). Spatial transcriptomic analysis of immunotherapy-resistant ESCC revealed that *COL11A1*+ CAFs are predominantly localized at the invasive front, forming a dense, collagen-rich peritumoral band that physically segregates CD8+ T cells accumulating in the stromal compartment from the tumor core. Multiplex immunofluorescence confirmed a 2.3-fold enrichment of *COL11A1*+ CAFs at tumor borders in non-responders compared with responders, establishing these cells as master regulators of immune-exclusive barrier formation (12). Although the direct contribution of COL11A1^+^ CAFs to elevated IFP has not been formally quantified in ESCC, excessive collagen deposition and cross-linking by CAFs is known to compress intratumoral blood and lymphatic vessels, increase interstitial solid stress, and impair fluid drainage—biomechanical processes that collectively drive IFP elevation (13).

The dense physical barrier formed by CAFs restricts interstitial fluid flow, leading to pressure imbalance across the tumor. Tumor cells also secrete pro-angiogenic factors such as VEGF. These factors promote structurally abnormal, hyperpermeable blood vessels. These vessels lack pericyte coverage and intact endothelial junctions. As a result, plasma proteins leak into the interstitium and fluid accumulates. This results in plasma protein extravasation and interstitial fluid accumulation. Furthermore, the absence of mature functional lymphatic vessels within the TME prevents effective fluid drainage. Under the combined influence of these three factors, the interstitial fluid pressure (IFP) rises dramatically from a normal range of −3 to +3 mmHg to as high as 10–100 mmHg. Since high-molecular-weight drugs such as ICIs are transported into tumors primarily via convection along hydrostatic pressure gradients, elevated IFP attenuates or even reverses this pressure gradient, thereby impairing ICI delivery (13, 14). Direct measurements of IFP in ESCC patient samples are scarce. but the biomechanical principles described above have been validated in other solid tumors, and the dense desmoplastic stroma observed in ESCC strongly suggests a similarly elevated IFP. Moreover, elevated IFP not only impedes the infiltration of antigen-specific T cells but also upregulates *TGF-β* expression in tumor cells, which in turn recruits Treg cells and suppresses CD8^+^ T cell function (15). The causal link between elevated IFP and Treg recruitment has been experimentally demonstrated in breast and colorectal cancer models (Level 3); its direct validation in ESCC remains an open area of investigation.

### 2.2 Enrichment of immunosuppressive cells

The immune cell landscape of the ESCC TIME is dominated by immunosuppressive populations, primarily regulatory T cells (Tregs), M2-type tumor-associated macrophages (M2-TAMs), and tolerogenic dendritic cells (tolDCs) (16, 17).

Tregs are a distinct subset of CD4+ T cells, accounting for approximately 5–10% of CD4+ T cells, characterized by high expression of the transcription factor FoxP3 and the IL-2 receptor alpha chain (CD25) (18). They secrete inhibitory cytokines such as transforming growth factor-β (*TGF-β*), IL-35, and IL-10, modulate the expression of inhibitory receptors, and promote intratumoral T cell exhaustion. In the ESCC TME, Tregs are not only more abundant than in adjacent normal tissues but also harbor a “hyper-Treg” subpopulation with enhanced suppressive activity, which interacts with progenitor CD8+ T cells (precursor exhausted CD8+ T cells with regenerative potential) through the CLEC2C-KLRB1 axis, thereby eliminating effector T cells at their source (19). Tumor-associated macrophages differentiate into two distinct subtypes: M1-TAMs enhance anti-tumor immunity by secreting IL-12 and TNF-α and directly killing tumor cells, whereas M2-TAMs promote immune escape by secreting *TGF-β1*, upregulating PD-L1, and directly interfering with T cell trafficking (20).After ICI therapy, tissue-resident macrophages (RTM-TAMs) increase in number and engage CD8^+^ T cells through a pseudo-antigen presentation mechanism involving MHC-I molecules, which, instead of activating effector T cells, induces T cell apoptosis (21). Dendritic cells (DCs) orchestrate anti-tumor immune responses by presenting tumor antigens to and activating T cells in tumor-draining lymph nodes (TDLNs). However, within the TME, some DCs are converted into tolDCs. Rapid tumor proliferation renders the tumor core severely hypoxic, driving glycolysis and abundant lactate production. When taken up by DCs, lactate activates the cholesterol synthesis regulator SREBP2 (sterol regulatory element-binding protein 2) which initiates the mevalonate pathway, driving the differentiation of DCs into mature regulatory DCs (a subtype of tolDC) that highly express CD63, which can directly inhibit CD8+ T cells and promote Treg generation (22). This lactate–SREBP2–tolDC axis was primarily elucidated in melanoma models (Level 3). Although lactate accumulation is a hallmark of ESCC, the contribution of this specific pathway to tolDC generation in the esophageal TME has not yet been experimentally confirmed.

### 2.3 Effector cell exhaustion and dysfunction

CD8+ T cells are principal effector cells of anti-tumor immunity. However, within the TME of ESCC, they are universally driven into a functionally impaired, exhausted state due to persistent antigen stimulation, the immunosuppressive microenvironment (Tregs, M2-TAMs, tolDCs), upregulation of checkpoint molecules, high expression of the transcription factor *TOX*, small extracellular vesicles carrying PD-L1 (sEV-PDL1), chromobox protein 6 (CBX6), and other factors (17, 23–25). Single-cell and functional studies in ESCC have directly mapped the trajectory of T cell exhaustion within the esophageal TME. Zheng et al. described the continuous evolution of CD8+ T cells from “pre-exhausted” to “exhausted” states in ESCC, where terminally exhausted CD8+ T cells highly express multiple immune checkpoint molecules, including *PDCD1 (PD-1)*, *TIGIT*, *CTLA-4*, *HAVCR2 (TIM-3)*, and *LAG-3*. Although they still express granzymes such as GZMB, their effector function is potently suppressed, and due to epigenetic changes, this state is relatively stable and more difficult to reverse by PD-1 blockade. Similarly, exhausted CD4+ T cells highly expressing *PDCD1, TIGIT, CXCL13, CCL5, CCL4, IFNG*, *CXCL1*, *TGF-β*and *TOX*, as well as NK cells highly expressing inhibitory receptors such as KLRC1 (NKG2A) and ITGA1 (CD49d), have been identified, and these three types of exhausted effector cells constitute the main proliferative population within ESCC tumors (17). Additionally, in patients with immunotherapy-resistant esophageal cancer, although germinal center B cells (GCBs) are enriched, their function is disrupted by tumor cells that highly express MIF. Through the MIF–CXCR4 axis, this prevents their normal differentiation into plasma cells, thus impairing humoral immunity (26).

### 2.4 Impaired antigen presentation and immune escape

Multiple independent analyses of ESCC resection specimens have demonstrated that approximately 40% of primary lesions and 90% of metastatic lesions in ESCC exhibit downregulation or loss of major histocompatibility complex (MHC) class I molecules, a rate significantly higher than that in other cancer types (27). The downregulation of MHC class I molecule expression reduces the ability of CD8+ T cells to recognize tumor antigens, facilitating tumor immune escape. Within the TME, PD-L1 expressed on tumor cells binds to PD-1 on T cells, rendering T cells dysfunctional, anergic, and exhausted, thereby preventing them from recognizing and killing tumor cells. In the ESCC TME, the situation is even more complex and severe: PD-L1 on esophageal cancer cells is often co-expressed with inhibitory molecules such as IDO1 and B7-H4, forming a synergistic inhibitory network that promotes tumor immune escape (28, 29). More recently, the co-expression landscape of immune checkpoints in ESCC has been further expanded: for example,CD39 and PD-L1 co-expression on stromal cells was associated with enhanced response to neoadjuvant immunotherapy (30), suggesting that multiple inhibitory axes may simultaneously shape the immunosuppressive milieu in this disease. Furthermore, ESCC cells can actively alter their immunophenotype to evade immune surveillance. For example, high expression of CD276 (B7-H3) drives the formation of neutrophil extracellular traps through the CXCL1-CXCR2 axis, which in turn suppresses NK cell function and facilitates immune escape (31).

As shown in **Figure 1**, these four mechanisms collectively constitute the microenvironmental basis for immunotherapy resistance in ESCC. Radiotherapy has the potential to simultaneously disrupt physical barriers, eliminate immunosuppressive cells, reverse T cell exhaustion, and upregulate MHC class I molecules, thereby addressing multiple facets of resistance.

**Figure 1:**
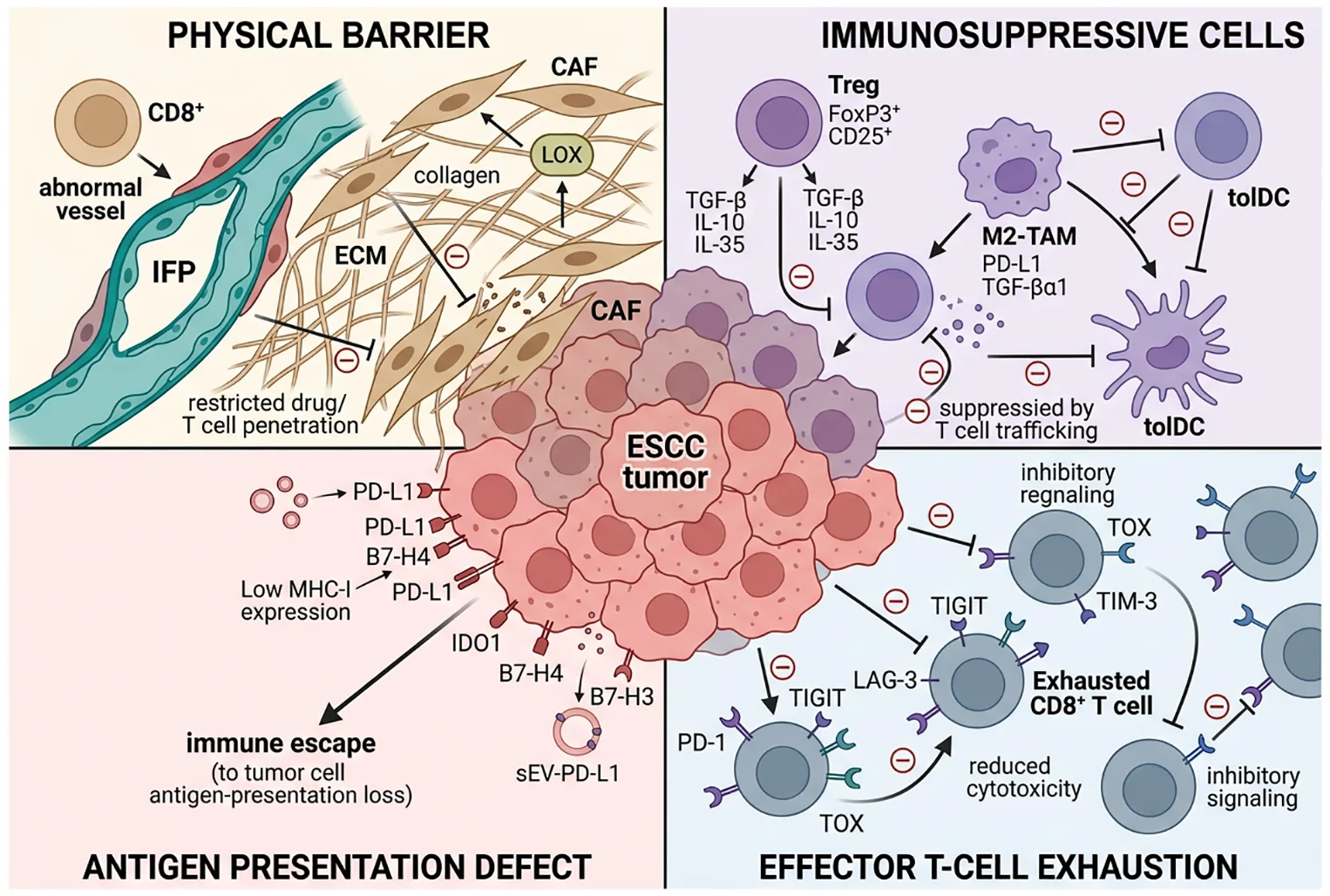
Four microenvironmental barriers driving immunotherapy resistance in ESCC. (1) Physical barrier: *COL11A1*^+^ CAFs deposit dense collagen that physically excludes CD8^+^ T cells and is hypothesized to elevate IFP through vascular compression. (2) Immunosuppressive cells: Tregs, M2-TAMs, and tolDCs are enriched within the TME. (3) Effector cell exhaustion: persistent antigen stimulation upregulates multiple checkpoint molecules (*PD-1, TIM-3, LAG-3, TIGIT, CTLA-4*) a, driving CD8^+^ T cell dysfunction. (4) Impaired antigen presentation: MHC class I downregulation and co-expression of PD-L1 with IDO1 and B7-H4 form a synergistic inhibitory network.

## 3 Core mechanisms of radiotherapy reversing immunotherapy resistance

As shown in **Figure 2**, we propose that radiotherapy may help overcome immunotherapy resistance through four coordinated mechanisms: remodeling physical barriers, eliminating immunosuppressive cells, reversing T cell exhaustion, and restoring tumor visibility. This can convert an immunologically ‘cold’ tumor microenvironment into a more inflamed state, thereby creating conditions that allow immune checkpoint inhibitors (ICIs) to function.

**Figure 2:**
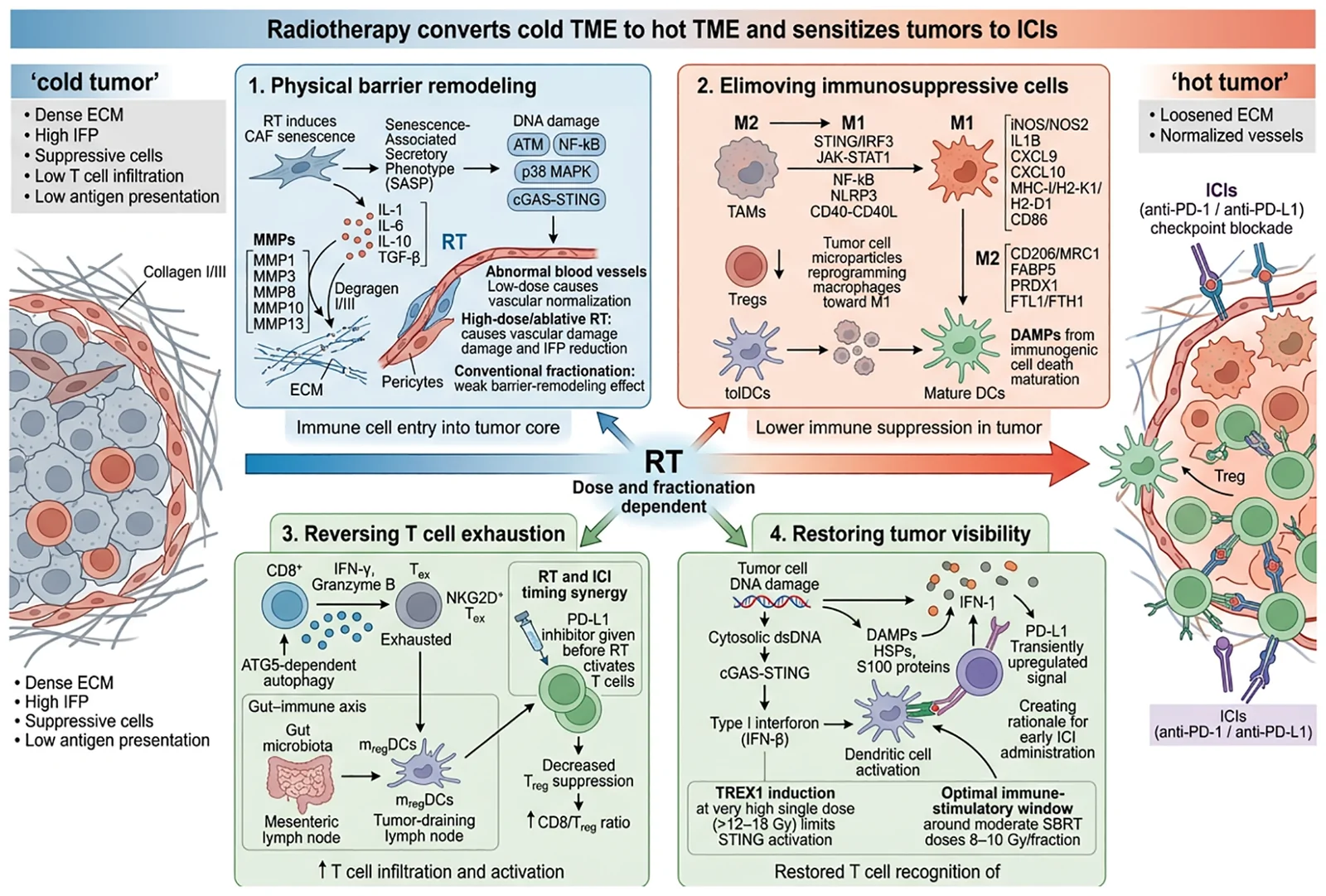
Four proposed mechanisms by which radiotherapy may help overcome immunotherapy resistance in ESCC. (1) Physical barrier remodeling: high-dose radiotherapy induces CAF senescence and *MMP*-mediated ECM degradation; low-dose radiotherapy normalizes vasculature and reduces IFP; SBRT decreases tumor cell density. (2) Immunosuppressive cell elimination: moderate-dose radiotherapy repolarizes M2-TAMs toward M1 phenotype; high-dose radiotherapy reduces Tregs; ICD matures tolDCs via DAMPs. (3) T cell exhaustion reversal: SBRT preserves intratumoral CD8^+^ T cells; optimized immunotherapy-before-radiotherapy sequencing enhances T cell reactivation. (4) Immune recognition restoration: SBRT at 8–10 Gy/fraction activates cGAS-STING, upregulates MHC class I, and transiently increases PD-L1.

### 3.1 Radiotherapy reshapes the tumor physical barrier: from CAF senescence and ECM degradation to IFP reduction

The physical barrier in the TME consists of two structural elements: the dense extracellular matrix (ECM) deposited by CAFs, and the elevated IFP by abnormal blood vessels. Together, these components exclude immune effector cells and antibody drugs from the tumor. Radiotherapy can disrupt this barrier at both levels simultaneously, but its effectiveness is strongly dependent on radiation dose and fractionation regimen.

#### 3.1.1 Radiation-induced CAF senescence and ECM degradation: opening the matrix barrier

CAFs are intrinsically radioresistant: rather than undergoing rapid apoptosis after irradiation, they undergo irreversible cellular senescence following DNA damage signals (such as ATM/NF-κB, p38 MAPK, and cGAS-STING). These senescent CAFs adopt the senescence-associated secretory phenotype (SASP), characterized by sustained secretion of pro-inflammatory and fibrotic factors (e.g., IL-1, IL-6, IL-10, TGF-beta), upregulation of multiple matrix metalloproteinases (MMPs) and suppression of type I and III collagen synthesis. The net effect is a shift in the ECM degradation–synthesis balance toward catabolism (32).

The “collagenolytic phenotype” of senescent fibroblasts — featuring robust MMP induction and diminished collagen production — was first characterized in irradiated breast and lung fibroblasts (33–35). Mavrogonatou et al. comprehensively reviewed this ECM remodeling program: across replicative senescence and stress-induced premature senescence (including irradiation and oxidative stress), *MMP1, MMP3, MMP8, MMP10*, and *MMP13* are consistently upregulated, endogenous inhibitors such as TIMP-2 are downregulated, and COL1A1/COL3A1 mRNA and total collagen synthesis are markedly reduced. Direct evidence in esophageal fibroblasts remained absent until recently (Level 3). Sugimoto et al. (2024) have used human esophageal mucosal fibroblast cells to induce cellular senescence (SA-Beta-gal positive, upregulation of p21/p16) and NF-κB activation of a single dose of 8 Gy; the supernatant of the culture medium causes proliferation, migration, invasion, and epithelial-mesenchymal transition (EMT) of the esophageal squamous carcinoma cells, and this effect is partially reversed by the NF-κB inhibitor metformin. Even though the study did not directly test the levels of the MMPs, the typical role of NF-κBin controlling the expression of genes encoding the matrix metalloproteinases and the overall increase in the levels of MMPs in SASP make it plausible that CAFs exposed to radiation will break down the ECM through the pathway of NF-κB -MMP (36). Although MMP levels were not directly measured in that study, the pronounced NF-κB activation and the established role of NF-κB in MMP gene transcription strongly support a senescence-driven ECM-degrading program in irradiated esophageal fibroblasts.

Moreover, single-cell and spatial transcriptomics of Yang et al. identified an *MMP1* + fibroblast subpopulation in treatment-naive esophageal squamous cell carcinoma tissues. This subpopulation is mainly enriched in the tumor core and functions to degrade ECM. More importantly, TGF- and IL-6 regulate its activation strictly in the microenvironment- both are major factors that are secreted abundantly by senescent CAFs through SASP following irradiation. *In vitro* co-culture experiments directly demonstrated that tumor cells stimulate fibroblasts to upregulate *MMP1* and *MMP3* via TGF-B/IL-6 signaling pathway, creating a positive feedback loop of matrix degradation (37).” These findings provide esophagus-specific histological support for the cascade: radiotherapy → CAF senescence → SASP (TGF-β/IL-6) → MMP1^+^ phenotype → ECM degradation.

Thus, while the initial characterization of radiation-induced CAF senescence relied on breast and lung fibroblast models, subsequent studies using human esophageal fibroblasts and ESCC tissue specimens have provided esophagus-specific support for this mechanism. Collectively, these data strongly suggest that high or ablative doses of radiotherapy can drive esophageal CAFs into irreversible senescence, thereby unleashing SASP-NF-κB-MMP-mediated ECM degradation, increasing matrix porosity (13), and creating corridors for immune cell infiltration and therapeutic antibody penetration into the tumor.

#### 3.1.2 Low-dose vascular normalization and high-dose ablation: dual pathways for reducing IFP

For the purpose of this review, low-dose radiotherapy (LDRT) refers to fraction sizes ≤2 Gy with total doses typically ranging from 10 to 40 Gy; stereotactic body radiotherapy (SBRT) refers to hypo-fractionated treatment delivered in 1–5 fractions with fraction sizes ≥5 Gy; and conventional fractionation refers to 1.8–2 Gy per fraction delivered over several weeks. The second component of the physical barrier is elevated IFP, which operates in concert with the ECM to impede drug delivery and immune cell infiltration. Radiotherapy has at least two ways of lowering IFP: preclinically (Level 3),low-dose radiation promotes vascular normalization by suppressing VEGF, enhancing pericyte coverage, and reducing vascular leakiness, thereby restoring tissue perfusion and lowering IFP (38). SBRT uses high single doses, typically ≥15 Gy. These doses kill tumor cells directly, relieve vascular compression, and damage endothelial cells. In murine breast cancer models(Level 3), SBRT reduced IFP by approximately 50% within 24 hours (39). Although such measurements have not been performed specifically in ESCC, the physical principle of vascular collapse relief by tumor debulking are likely applicable.

Nevertheless, this effect is not possible with all fractionation schedules. In a mouse model of human esophageal cancer(Level 3), conventional fractionation (2Gy x 5 fractions/week) administered continuously over 2 or 4 weeks did not significantly alter tumor IFP (40). Although this study remains, to our knowledge, the only direct comparison of fractionation schedules on IFP in an esophageal cancer model, its findings are consistent with the principle—demonstrated in other tumor types (Level 3)—that clinically meaningful IFP reduction generally requires high single doses (39).This negative result implies that the intensity threshold required to induce CAF senescence-MMP pathways or sufficient vascular remodeling may be beyond the capability of conventional fractionation. However, this mechanistic inference has not been directly tested in clinical trials, and whether this IFP-sparing effect translates into impaired ICI delivery remains an open question.

At present, the fractionation of chemoradiotherapy in the neoadjuvant treatment of esophageal cancer is based on a conventional one with the most popular being CROSS (41.4 Gy in 23 fractions) (41). The addition of PD-1 inhibitors to this backbone has been evaluated in three phase II trials in ESCC, with inconsistent results. PALACE-1, a single-arm study combining pembrolizumab with the CROSS regimen, reported a pCR rate of 55.6% (10/18) (42). However, this early signal was not reproduced in larger cohorts. PALACE-2, the largest trial to date (n = 140), used a similar regimen of preoperative pembrolizumab plus chemoradiotherapy and achieved a pCR rate of 43.2%, which the authors concluded was not superior to historical outcomes with chemoradiotherapy alone (43). ACTS-29, which evaluated neoadjuvant pembrolizumab plus chemoradiotherapy (44.1 Gy in 21 fractions) followed by adjuvant pembrolizumab, reported a pCR rate of only 23.1%—falling short of the prespecified 30% threshold (44). Taken together, these trials indicate that adding a PD-1 inhibitor to conventionally fractionated chemoradiotherapy does not reliably improve pathologic response in ESCC. Whether this limited benefit reflects insufficient ECM degradation by conventional fractionation remains an untested hypothesis: none of these studies prospectively measured IFP, ECM components, or intratumoral drug penetration, and alternative explanations—such as differences in nodal burden, chemotherapy regimens, or the extent of lymphadenectomy—cannot be excluded. In the adjuvant setting, CheckMate-577 demonstrated that nivolumab prolonged disease-free survival after neoadjuvant chemoradiotherapy; the benefit was more pronounced in patients with squamous cell carcinoma than in those with adenocarcinoma, although it appeared independent of tumor-cell PD-L1 expression (5), Indicating that not all patients benefit equally. Together with the PACIFIC trial in locally advanced non-small cell lung cancer (Level 2)—where consolidative durvalumab after definitive chemoradiotherapy established the paradigm that radiation-induced immunogenic cell death can prime the immune system for subsequent checkpoint blockade (45)—these data underscore that the synergy between radiotherapy and immunotherapy is highly context-dependent. This apparent disconnect—whereby conventionally fractionated chemoradiotherapy can provide meaningful clinical benefit via direct cytoreduction yet fails to optimally synergize with immunotherapy—merits reconciliation. The CROSS regimen’s established efficacy is primarily attributable to the direct tumor-killing effects of chemotherapy and radiotherapy, which achieve local control and survival benefit largely independently of IFP reduction or immune reprogramming. The inconsistent pCR rates observed in the ESCC trials discussed above are consistent with, but do not prove, the possibility that conventional fractionation does not sufficiently open the ECM barrier or lower IFP when immunotherapy is added. In other words, while CROSS succeeds as a cytotoxic strategy, its capacity to remodel the physical barrier for subsequent immunotherapy has not been established—and this represents a gap that hypo-fractionated or ablative radiotherapy strategies may help address.

#### 3.1.3 Summary

Overall, radiotherapy-induced remodeling of the tumor physical barrier is strongly dose- and fractionation-dependent. Preclinical and early clinical evidence indicates that high-dose or ablative fractionation may be required to trigger CAF senescence-SASP-MMP-mediated ECM degradation and meaningful IFP reduction; Conventional fractionation only has minimal effects on both barriers-a fact that, while not negating the established cytotoxic benefits of standard chemoradiotherapy, could be one contributing factor among several when ICI is added. Whether this contributes to the modest pathologic response rates observed in chemo-immuno-radiotherapy trials remains an untested hypothesis; prospective measurements of IFP and ECM dynamics will be required to establish any causal relationship. The future research should address: (1) screening radiation parameter combinations capable of activating ECM degradation and keeping low interstitial pressure state at the same time; (2) combination of anti-fibrotic drugs (e.g., TGF-β inhibitors, FAK inhibitors) or SASP-modulating agents (e.g., metformin) to synergistically break down the physical barrier and further improve immunotherapeutic efficacy.

### 3.2 Eliminating immunosuppressive cells: disintegrating the immunosuppressive network

The effect of radiotherapy on immunosuppressive cells in the TME presents a typical “double-edged sword” effect: on one hand, conventional radiotherapy parameters can induce their accumulation, leading to treatment resistance; on the other hand, optimized radiotherapy regimens or combination strategies can effectively eliminate or weaken these cells.

For instance, the polarization effect of radiotherapy on tumor-associated macrophages (TAMs) is dose-dependent. These polarization patterns have been established primarily in murine tumor models and macrophage cell lines (Level 3); their direct replication in the ESCC microenvironment has not yet been formally tested. Both high-dose (>10 Gy/fraction) and very low-dose (<1 Gy/fraction) radiotherapy can promote TAM polarization toward the M2 phenotype, accompanied by the secretion of IL-10 and TNF-α, whereas moderate-dose (1–10 Gy/fraction) RT can promote TAM polarization toward the M1 phenotype (Level 3) (46, 47). Moreover, microparticles of tumor cells produced by radiotherapy can reprogram M2 TAMs into M1 TAMs through the JAK-STAT and MAPK pathways (Level 3) (48). Specifically, low-dose radiotherapy in combination with immunotherapy (e.g. RACIM regimen) (Level 2)creates a complex multi-pathway synergistic network that leads to bidirectional gene reprogramming of M2-like macrophages at the transcriptomic level, ultimately transforming them into tumoricidal M1-like macrophages (38). In preclinical models (Level 3), LDRT first causes DNA damage and type I interferon response within tumor cells thus triggering the STING/IRF3/IRF8 and JAK-STAT1 signaling axes which create a permissive inflammatory milieu. Based on this premise, co-administered immunotherapy unleashes and expands polyfunctional CD4+ and CD8+ effector T cells, which serve as a long-term source of IFN-γ. Simultaneously, enhanced CD40-CD40L signaling interacts synergistically with NF-κB and NLRP3 inflammasome pathway directly enhancing pro-inflammatory gene programs. This synergy results in a deep remodeling of gene expression: on one hand, M1 signature genes are highly induced, such as the prototypical Nos2 (iNOS), the pro-inflammatory cytokine Il1b, the chemokines Cxcl9/Cxcl10, antigen-presentation molecules H2k1/H2d1, and co-stimulatory molecules Cd40/Cd86, a reprogramming pattern typical of the transcriptional features of classically activated macrophages in the tumor microenvironment (49, 50). Since this foundational characterization of M1/M2 polarization, radiotherapy-induced TAM reprogramming has been directly demonstrated in a murine ESCC model, where combining anti-PD-L1 with radiotherapy reduced M2-type TAMs and increased tumor-infiltrating CD8^+^ T cells (Level 3) (51). Moreover, single-cell and spatial transcriptomics have further refined TAM classification in ESCC beyond the M1/M2 dichotomy, identifying tumor-enriched SPP1^+^ macrophages as a dominant immunosuppressive subset that correlates with poor clinical outcomes (52). On the other hand, M2-associated genes are systematically down-regulated, such as the pattern-recognition receptor Mrc1 (CD206), the fatty acid-binding protein Fabp5 (53, 54), the genes involved in redox regulation and inflammatory mediator synthesis (e.g., Prdx1), and the ferritin genes Ftl1/Fth1 (55, 56). Moreover, genes involved in inflammation-related microenvironmental remodeling, including Tmsb4x, are also suppressed. At the same time, a metabolic shift towards glycolysis is observed, as evidenced by the increased expression of mitochondrial gene mt-Nd2, which provides bioenergetic needs of M1 effector functions and correlates with the known metabolic reprogramming of polarized macrophages (57). Altogether, these multi-layered transcriptional changes form a self-reinforcing positive-feedback loop: the reprogrammed M1-like macrophages release *CXCL9/CXCL10* to attract more T cells and present antigens effectively using upregulated MHC-I and CD86, whereas infiltrating T cells further consolidate the M1-polarized state by secreting IFN-. This orchestrated system, which includes epigenetic/transcriptional priming, metabolic remodeling, and immune cell cross-talk, not only triggers full activation of the M1 program but also eliminates maintenance signals of the M2 program, thus ensuring a complete transition of macrophages from a pro-tumoral M2 phenotype to a tumoricidal M1 phenotype (58). It should be noted, however, that the intricate gene expression changes described above were delineated in a single study using a syngeneic colorectal cancer model; independent replication of this specific LDRT-induced macrophage reprogramming program in ESCC has not yet been reported. While the RACIM regimen provides compelling proof of concept, the generalizability of its findings to the esophageal TME warrants further investigation.

As for Treg cells, evidence from prostate cancer models demonstrates that high-dose radiotherapy, alone or combined with ICI, can significantly reduce the intratumoral Treg fraction and increase the CD8/Treg ratio (Level 2) (59). In contrast, the LDRT-based RACIM regimen achieves Treg functional suppression indirectly—through M2→M1 macrophage reprogramming and enhanced IFN-γ production—rather than through direct depletion (38). These two approaches thus operate through distinct immunological mechanisms at different dose ranges, indicating that both high-dose and low-dose strategies can ultimately shift the Treg/Teff balance, albeit via different routes. Notably, extremely high total radiation doses (>30 Gy) can also abrogate Treg immunosuppressive function, likely through disruption of autophagy-mediated Treg survival(Level 3) (60). For tolDCs, radiotherapy alone can paradoxically enhance their capacity to induce Treg differentiation, thus promoting tumor progression; however, when radiotherapy combined with immunotherapy, it induces immunogenic cell death (ICD) and the release of damage-associated molecular patterns (DAMPs). Which can restore the function of tolerogenic DCs, leading to their functional maturation (Level 3) (61, 62).

### 3.3 Reversing T cell exhaustion: restoring effector cell function

In the TME of ESCC, CD8+ T cells are widely in a state of functional exhaustion. Radiotherapy can reverse this state through multiple pathways. In a murine esophageal cancer model(Level 3), SBRT showed a unique advantage in maintaining intratumoral CD8^+^ T cells, while conventional fractionation caused sustained depletion (63). First, by eliminating immunosuppressive cells, especially Tregs, radiotherapy releases the critical brake on T cell proliferation and effector function (64). Second, optimizing the timing of radiotherapy and immunotherapy is crucial. Research has shown that administering a PD-L1 inhibitor 3 days before radiotherapy can significantly increase the proportion of reactivated T cells within the tumor and reduce the proportion of exhausted T cells (Level 3) (65). Third, radiotherapy can induce novel T cell subsets with potent effector functions. Low-dose radiotherapy combined with immunotherapy can induce a large number of NKG2D+ exhausted T cells (Tex) with proliferative potential. Although these cells express exhaustion markers, they concurrently possess powerful effector functions (IFN-γ, GzmB) dependent on NKG2D signaling and are key to therapeutic efficacy (Level 2) (38). Whether this specific T cell subset can be reliably induced by LDRT in ESCC has not been independently assessed. Fourth, the modulating role of autophagy. ATG5-dependent autophagy is essential for T cell proliferation and functional maintenance, and radiotherapy-induced autophagy can promote CD8+ T cell infiltration (Level 3) (60). Finally, radiotherapy can occur via the gut–immune axis. Low-dose irradiation deliberately delivered to the gut (1 Gy) can reshape the gut microbiota (e.g., Christensenella minuta), promoting the migration of immunoregulatory molecules (mregDCs) from mesenteric lymph nodes to tumor-draining lymph nodes, thereby enhancing intratumoral T cell infiltration and function and reducing exhaustion (Level 2) (66).

### 3.4 Restoring tumor visibility: enhancing antigen presentation

Tumor cells evade immune recognition by downregulating MHC class I molecule expression, but radiotherapy can restore tumor cell “visibility” through multiple pathways. SBRT, owing to its high-dose characteristics, can more effectively activate the cGAS-STING pathway. Studies have shown that SBRT delivering 8 Gy per fraction can promote the release of double-stranded DNA (dsDNA) from tumor cells. Which activates the cGAS–STING signaling pathway, leading to type I interferon (IFN-β) production, dendritic cell (DC) activation, and subsequent priming of effector T cells (Level 3) (67, 68). However, the SBRT dose cannot be arbitrarily escalated. In breast and colon carcinoma models(Level 3), a single fraction exceeding 12–18 Gy can induce the expression of the DNA exonuclease *TREX1*, which rapidly degrades cytoplasmic DNA, thereby terminating cGAS-STING signaling and weakening the immune effect (68). The immunogenicity of SBRT depends on dose selection – moderate doses (8–10 Gy per fraction) can activate innate immunity, whereas single extremely high doses (>15 Gy) actually weaken the immune response due to *TREX1* activation. Extending these principles to ESCC, radiation-induced MHC-I upregulation and transient PD-L1 expression have been confirmed in esophageal cancer cell lines and patient specimens, respectively (63, 69), suggesting that the same dose-dependent immune activation operates in esophageal tumors. However, the clinical translation of these immunologically optimal dose ranges must account for cumulative biological effective dose and normal tissue tolerance, particularly given the central thoracic location of the esophagus and its proximity to multiple organs at risk; the feasibility and safety of such regimens in patients with ESCC require dedicated prospective evaluation.

Radiation induces a dose-dependent upregulation of MHC class I expression, peaking at 8 Gy (Level 3). Radiotherapy can also upregulate MHC-I through an indirect pathway mediated by IFN-γ. Radiotherapy does not directly stimulate tumor cells to produce IFN-γ; instead, by damaging tumor cells, it releases a series of DAMPs such as heat shock proteins (HSPs) and S100 proteins, thereby activating infiltrating immune cells (e.g., NK cells, NKT cells) to secrete IFN-γ (69, 70). IFN-γ increases MHC-I expression and enhances antigen presentation through the JAK/STAT signaling pathway (Level 3) (71, 72). Notably, IFN-γ only increases the proportion of T cells but does not induce their activation; it is the radiation that serves as the activating factor for T cells (69). Additionally, radiotherapy can promote the maturation and antigen-presenting function of DCs by inducing ICD and releasing tumor-specific antigens (Level 3) (73). Finally, radiotherapy can upregulate PD-L1 expression, thereby enhancing the efficacy of PD-L1 inhibitors, an effect primarily associated with the activation of the DNA damage response and the cGAS-STING signaling pathway(Level 3) (74). In ESCC, radiotherapy can also upregulate PD-L1 through the IL-6/STAT3 signaling pathway. Further research has revealed that this PD-L1 upregulation is markedly transient, peaking at 48–72 hours after irradiation and returning to baseline after 7 days. This suggests that administering ICIs as early as possible after radiotherapy may achieve optimal synergistic effects (75).

## 4 Discussion

The relationship between radiotherapy and the immune system has traditionally been described in binary terms—immunostimulatory versus immunosuppressive—but the systematic analysis presented in this review suggests a more nuanced view. The immune effects of radiotherapy depend on the combination of multiple parameters, including dose, fractionation schedule, irradiation field, and timing of combination, thus representing a precisely controllable therapeutic modality. Based on current evidence, radiotherapy reversal of immunotherapy resistance in esophageal cancer can be integrated into a three-step progressive model: first, radiotherapy, especially SBRT, induces ICD to release tumor-specific antigens, activating DCs and naïve T cells; second, radiotherapy, including low-dose RT, remodels the TME by disrupting physical barriers, reducing IFP, eliminating immunosuppressive cells, and upregulating PD-L1 and MHC class I molecules, directly promoting effector T cell infiltration and facilitating ICI action; finally, immune checkpoint inhibitors such as anti-PD-L1 act on this basis to promote the expansion of effector T cells and exert synergistic anti-tumor effects. This three-step model illustrates that radiotherapy and immunotherapy cooperate through the convergence of multiple, temporally distinct mechanisms.

### 4.1 Radiotherapy fractionation strategies: SBRT versus LDRT

The optimal radiotherapy schedule for ICI-resistant ESCC remains unclear. Available studies use a wide range of doses and fractionations. These range from LDRT (2 Gy × 10 fractions) to SBRT (8 Gy × 3–5 fractions). No head-to-head comparison of these approaches has been conducted in patients with ICI-resistant ESCC. All discussions regarding the relative merits are therefore based on indirect evidence and cross-trial comparisons, which should be interpreted with caution. This section examines the mechanistic rationale and practical considerations that may guide the choice between these two strategies, while the corresponding clinical evidence is critically evaluated in Section 4.2.

Preclinically (Level 3), the mechanistic rationale for SBRT rests on the hypothesis that high-dose radiation induce ICD and release a large quantity of tumor-specific antigens to activate effector T cells, making it particularly suitable for managing oligometastatic resistant lesions. Although this hypothesis is supported by preclinical data, it has not been directly tested in prospective ESCC trials. Its limitations include: first, due to the unique anatomical location of the esophagus, high-dose radiation combined with immunotherapy significantly increases the risk of associated adverse events, such as pneumonitis (including radiation pneumonitis and immune-related pneumonitis) and cardiotoxicity; second, regarding the timing of combination, administering immunotherapy within 1 week after radiotherapy may yield better efficacy, but the risk of overlapping toxicity requires careful evaluation. Preclinically (Level 3), the advantages of LDRT lie in its modulation of the TME: it can reduce IFP, improve T cell infiltration, decrease the abundance of M2-type macrophages while minimizing lymphocyte depletion. It may be more tolerable for patients with poor performance status and potentially associated with lower toxicity. Its limitation lies in the restricted capacity for direct tumor cell killing and ICD induction.

Beyond these strategic questions, several practical considerations must be addressed when implementing radiotherapy-immunotherapy combinations in esophageal cancer patients. First, target volume delineation requires careful differentiation between the primary esophageal lesion and metastatic lymph nodes; respiratory motion management and individualized PTV margins are essential to minimize toxicity while ensuring adequate target coverage. Second, strict adherence to organ-at-risk dose constraints is critical given the central thoracic location of the esophagus. Specific constraints vary according to fractionation scheme, target location, and institutional practice; current guidelines recommend individualized planning that accounts for cumulative doses from prior thoracic radiation and the proximity of critical structures such as the heart, lungs, and spinal cord. Third, while preclinical evidence suggests that administering ICIs within one week of radiotherapy may optimize synergy, the optimal sequencing in the post-resistance setting has not been prospectively defined and must be weighed against the risk of overlapping toxicities, particularly pneumonitis. Finally, patient selection should incorporate performance status, prior radiation history, pre-existing autoimmune conditions, and baseline pulmonary and cardiac function; these factors may be particularly important when considering SBRT-based regimens, which carry a higher risk of organ-specific toxicity. These practical issues, although beyond the scope of the mechanistic focus of this review, are critical for the safe and effective translation of radio-immunotherapy strategies into clinical practice.

Nevertheless, the following critical questions in the field of esophageal cancer still need to be addressed by rigorously designed clinical trials: ① In patients with oligometastatic resistance, is SBRT superior to LDRT? ② In patients with widespread metastases, could LDRT be the preferred option for “immune sensitization”? ③ Is the combined application of both modalities (e.g., using SBRT to treat the dominant lesion and release antigens, followed by LDRT to modulate the overall microenvironment) superior to a single modality?

An important caveat in cross-study comparisons is that SBRT and LDRT have typically been evaluated in different clinical contexts—SBRT predominantly in oligometastatic patients with low tumor burden and good performance status, whereas LDRT has more often been applied to refractory patients with higher disease burden after multiple lines of therapy. This discrepancy in baseline patient characteristics precludes direct comparison of the survival outcomes reported in individual studies. The clinical evidence currently available for each strategy, and the critical gaps that remain, are systematically examined in the following section.

### 4.2 Clinical evidence of radiotherapy combined with immunotherapy in ICI-resistant ESCC

#### 4.2.1 Evidence supporting SBRT for oligometastatic resistant disease

The key clinical studies evaluating radiotherapy combined with immunotherapy in ICI‑resistant ESCC are summarized in **Table 1**. A key hypothesis raised in Section 4.1 is that SBRT, by virtue of its potent ICD induction and antigen release, should be preferentially effective for oligometastatic resistant disease. The most direct observational evidence to date comes from Liu et al., who reported the largest multicenter retrospective analysis to date (n = 335) and introduced a novel stratification by progression pattern: in patients with oligo-progression after ICI failure, radiotherapy significantly prolonged both PFS (9.4 vs 5.8 months) and OS (21.9 vs 9.2 months), whereas those with poly-progression derived no significant benefit (76). This retrospective observation is consistent with, but does not establish, a causal role for physical barrier disruption or antigen release in the observed clinical benefit; IFP and ECM parameters were not measured.

**Table 1:** Clinical and ongoing prospective studies evaluating radiotherapy combined with immunotherapy in immunotherapy-resistant esophageal squamous cell carcinoma.

Study	Design	Population	Intervention	Primary endpoint	Key outcomes	Ref
Zhao et al., 2023	Phase II, single-arm	Oligometastatic ESCC after 1L ICI + chemo failure	LDRT (30 Gy/10 fr or 40 Gy/20 fr) + camrelizumab + irinotecan	PFS	mPFS 6.9 mo; mOS 12.8 mo; ORR 40.8%	(77)
Liu et al., 2025	Retrospective, multicenter	Metastatic EC after 1L ICT failure	RT (IMRT/SBRT) ± IO rechallenge	PFS, OS	Oligoprogression: RT improved PFS (9.4 vs 5.8 mo) and OS (21.9 vs 9.2 mo)	(76)
Song et al., 2026	Retrospective, comparative	Metastatic ESCC after 1L IO failure	RT + IO re-challenge + chemo vs CRT alone	PFS, OS	mOS 11.4 vs 6.2 mo (HR 0.48); mPFS 6.8 vs 3.9 mo	(78)
ChiCTR2600121031	Phase II, single-arm	Advanced unresectable/metastatic ESCC after progression on anti-PD-(L)1	Cadonilimab + involved-field IMRT (50–60 Gy/25–30 fr) or SBRT to oligometastases (4–8 Gy/fr, 3–5 fr)	ORR	Pending	(79)
NCT07164690	Phase II, single-arm	Advanced ESCC resistant to 1L chemo-immunotherapy	LDRT single 2 Gy to all visible metastatic lesions + continued prior anti-PD-(L)1	PFS at 1 year	Pending	(80)

Studies were included only if the enrolled population predominantly comprised patients with prior anti–PD-(L)1 exposure and subsequent disease progression. Trials without published efficacy results are listed by registry number and marked as “Pending”.

1L, first-line; 2L, second-line; CRT, chemoradiotherapy; EC, esophageal cancer; ESCC, esophageal squamous cell carcinoma; fr, fractions; HR, hazard ratio; ICI, immune checkpoint inhibitor; ICT, immunochemotherapy; IMRT, intensity-modulated radiotherapy; IO, immunotherapy; LDRT, low-dose radiotherapy; mOS, median overall survival; mPFS, median progression-free survival; ORR, objective response rate; PFS, progression-free survival; RT, radiotherapy; SBRT, stereotactic body radiotherapy.

#### 4.2.2 Evidence supporting LDRT for microenvironmental modulation

Conversely, Section 4.1 proposed that LDRT may be primarily suited for patients in whom TME modulation—rather than direct cytoreduction—is the therapeutic goal. Zhao et al. directly tested this strategy in a single-arm phase II trial enrolling oligometastatic ESCC patients who had objectively progressed on first-line immunochemotherapy. Low-dose radiotherapy (30 Gy/10 fractions or 40 Gy/20 fractions) combined with camrelizumab and irinotecan achieved a median PFS of 6.9 months, a median OS of 12.8 months, and—notably—an abscopal response rate of 34.7% in unirradiated lesions (77). This abscopal signal suggests that LDRT can generate systemic immune effects beyond the irradiated field, directly aligning with the TME-remodeling rationale outlined in Section 4.1. However, abscopal responses in this trial were assessed by imaging rather than by direct measurement of IFP or ECM remodeling, and the mechanistic link between LDRT and the observed systemic effects remains indirect.

#### 4.2.3 Real-world evidence and its limitations

One additional retrospective comparative studies provide real-world corroboration in a population more precisely defined by prior immunotherapy exposure. Song et al. reported consistent findings in a direct comparison of ICI re-challenge plus chemoradiotherapy versus chemoradiotherapy alone (mOS 11.4 vs 6.2 months; HR 0.48) (78). This study, however, remains retrospective, radiotherapy regimens varied, and the analysis did not stratify patients by dominant resistance phenotype.

#### 4.2.4 Evidence gaps and ongoing trials

Despite these encouraging signals, the evidence base has critical deficiencies that limit the translation of the mechanistic framework proposed in Section 4.1 into clinical practice. First, no completed trial has directly compared SBRT with LDRT in ICI-resistant ESCC; the central question of whether the barrier-breaking or TME-modulating strategy should take precedence therefore remains unanswered. Second, all completed studies are single-arm or retrospective, precluding definitive conclusions about the incremental benefit of radiotherapy over ICI re-challenge alone. Third, the absence of biomarker-stratified analyses means that patients most likely to benefit from each strategy cannot yet be prospectively identified.

Two prospectively registered phase II trials directly targeting this population have recently appeared in the trial registries. ChiCTR2600121031, which has completed recruitment, is evaluating the PD-1/CTLA-4 bispecific antibody cadonilimab combined with involved-field or SBRT radiotherapy in patients with advanced ESCC who have progressed on prior anti–PD-(L)1 therapy; its primary endpoint is ORR, but no efficacy results have been reported (79). NCT07164690, not yet recruiting, will evaluate single-fraction low-dose radiotherapy (2 Gy) to all visible metastatic lesions followed by continuation of the prior anti–PD-(L)1 inhibitor in patients with progression after first-line chemo-immunotherapy, using 1-year PFS as the primary endpoint (80). These trials represent the first prospective efforts designed specifically to evaluate radiotherapy combined with immune checkpoint blockade in the post-resistance setting, and their results—when available—will provide critical information on whether the mechanistic hypotheses outlined in this review can be translated into measurable clinical benefit. Trials specifically designed to randomize ICI-resistant patients between SBRT-based and LDRT-based strategies, with prospective biomarker assessment including the barrier-exhaustion score proposed in this review, remain an urgent unmet need.

Finally, it is worth noting that the preclinical evidence base for fractionation-dependent immunological effects, while not derived from esophageal cancer models, is mechanologically consistent with the clinical observations discussed above. Comparative studies in syngeneic mouse models have shown that, under equivalent biologically effective doses, ablative hypo-fractionated radiotherapy more effectively suppresses tumor recruitment of myeloid-derived suppressor cells and enhances CD8^+^ T cell-mediated cytotoxicity compared with conventional fractionation (81); *in vitro* data further suggest that cells surviving conventional fractionation acquire a more aggressive phenotype than those surviving hypofractionation (82). However, as emphasized by a recent NCI consensus statement, no single fractionation schedule is universally optimal for immunomodulation; the choice must be guided by tumor characteristics, the specific immunotherapy used, and the therapeutic goal (83). Taken together, these data support the existence of dose- and fractionation-dependent differences in the immunological effects of radiotherapy, while underscoring that direct clinical evidence for the superiority of one strategy over another in reversing ICI resistance in esophageal cancer remains absent—a gap that the barrier-exhaustion score and future biomarker-stratified trials may help address.

### 4.3 Individualized therapeutic strategies guided by dominant resistance mechanisms: from cross-cancer insights to implications for ESCC

For ESCC patients who have developed ICI resistance, the selection of subsequent therapeutic strategies should be guided by the dominant resistance mechanism. This concept has been indirectly validated in other cancer types with distinct resistance features. The cross-cancer comparisons that follow are intended as conceptual analogies: the mechanisms they illustrate have been validated in their respective tumor types, but their applicability to ESCC—which likely harbors a more complex, mixed TME—remains to be formally demonstrated.

For resistance dominated by physical barriers, pancreatic ductal adenocarcinoma represents the most classic model. Its hallmark pathological feature is an abundant CAF-derived stroma and markedly elevated IFP, together forming a nearly impermeable barrier that excludes effector T cells from the tumor core. In this context, the core therapeutic logic is “breaking the barrier”— degrading the ECM and lowering IFP to create corridors for T cell infiltration. SBRT or LDRT can directly degrade ECM components and reduce IFP, and when combined with anti-angiogenic agents, can further normalize abnormal tumor vasculature and synergistically improve drug delivery. Notably, ESCC similarly harbors a dense collagen barrier formed by COL11A1^+^ CAFs at the invasive front, with spatial distribution features closely paralleling those observed in pancreatic cancer, suggesting that barrier-dominated ESCC may benefit from the same barrier-breaking strategy.

At the opposite end of the spectrum, melanoma and Clear Cell Renal Cell Carcinoma (ccRCC) provide instructive models of exhaustion-dominant resistance, although, as with pancreatic cancer, these parallels are drawn from well-studied tumor types whose TME features only partially overlap with those of ESCC. Melanoma usually has little physical barrier. T cells infiltrate the tumor abundantly. The major problem is T cell exhaustion. In these tumors, T cells are present but dysfunctional. ccRCC similarly displays rich T cell infiltration, yet due to *VEGF*-driven factors that establish a pan-tumor immunosuppressive TME, infiltrating T cells are locked in a state of deep exhaustion by multiple inhibitory receptors, including PD-1, TIM-3, and LAG-3, leading to both primary and acquired resistance. In these two cancer types, the central problem is no longer that “T cells cannot enter,” but that “T cells do not function.” Even if radiotherapy is applied to release neoantigens, effective immune responses may still fail to be initiated due to the coordinated suppression by multiple inhibitory receptors. Therefore, for such patients, the core therapeutic logic is “potent reinvigoration” or “bypass attack.” Bispecific antibodies (such as cadonilimab, simultaneously blocking PD-1 and CTLA-4) or novel ICI combination regimens can cover a broader spectrum of inhibitory signals; antibody–drug conjugates (ADC drugs),by precisely delivering cytotoxic payloads, can bypass the immune exhaustion machinery and directly kill tumor cells. Radiotherapy may serve a synergistic role as an “*in situ* vaccine,” providing an initial immune activation signal through the release of neoantigens.

These two cancer types serve as instructive extremes of barrier-dominant and exhaustion-dominant resistance, respectively—with the important caveat that neither fully recapitulates the ESCC microenvironment. ESCC likely occupies an intermediate position on this spectrum, harboring both a prominent collagen barrier and deeply exhausted T cells. The barrier-exhaustion score proposed in this review is therefore designed to capture this mixed phenotype rather than force a binary classification.

Extrapolating this framework to ESCC, the complexity lies in the potential coexistence of both physical barrier and immune exhaustion mechanisms within the same tumor. Therefore, predicting the dominant resistance mechanism in an individual patient becomes the key to selecting the optimal individualized therapeutic strategy. This is precisely where spatial transcriptomic biomarkers hold promise. As a working hypothesis, future work could aim to develop a conceptual “barrier-exhaustion score” system that integrates physical barrier integrity (e.g., the abundance and spatial distribution of COL11A1^+^ CAFs) and the degree of immune exhaustion (e.g., the co-expression gradient of multiple inhibitory receptors on CD8^+^ T cells). This score is currently a research instrument rather than a clinical decision aid: its construction, validation, and clinical applicability have not been established, and no prospective study has yet used it to stratify patients. Such a score could be constructed using existing spatial transcriptomic platforms that already enable simultaneous quantification of CAF abundance and T cell exhaustion markers within individual tumor sections; its prognostic value would first need to be tested retrospectively in cohorts of ICI-resistant ESCC with known treatment outcomes, and if validated, could then be applied prospectively to stratify patients in future radio-immunotherapy trials. Should such a score be developed and validated, patients with high barrier scores might preferentially receive barrier-breaking strategies, such as radiotherapy combined with anti-angiogenic agents; those with high exhaustion scores could be more suitable for potent reinvigoration or bypass attack strategies, such as bispecific antibodies or ADCs, with radiotherapy added as needed to provide synergistic antigen release. This hypothesis-driven, spatially informed framework remains entirely experimental and, if confirmed, may in the future help shift salvage therapy for ESCC from empirical trial-and-error toward biomarker-guided precision medicine.

## 5 Concluding remarks and future directions

Radiotherapy is uniquely positioned to address the multiple facets of ICI resistance in esophageal cancer. By simultaneously targeting the physical barrier (through CAF senescence -mediated ECM degradation and IFP reduction), the immunosuppressive cellular milieu, and the impaired antigen presentation machinery, it has the potential to contribute to the conversion of an immunologically ‘cold’ tumor microenvironment into a more inflamed, treatment-responsive state. The efficacy of this approach appears to depend critically on dose and fractionation. While conventional fractionation retains its established efficacy for tumor cytoreduction and local control, alternative dose and fractionation strategies may provide additional immunomodulatory effects that are particularly relevant when radiotherapy is combined with checkpoint inhibition. Prospective studies that directly measure IFP, ECM dynamics, and drug penetration are needed to confirm or refute this hypothesis. Ablative regimens are associated with more robust antigen release and oligo-metastasis control in early-phase studies; low-dose radiotherapy shows promise in stroma remodeling with less lymphotoxicity. The best choice depends on the dominant resistance phenotype. Spatial transcriptomics now enables simultaneous quantification of barrier integrity and T-cell exhaustion depth. The hypothetical “barrier-exhaustion” scoring system proposed in this review, although yet to be developed and validated, may in the future help guide mechanism-driven individualized radio-immunotherapy. We propose that future phase II trials adopt an adaptive design in which patients are stratified by a preliminary barrier-exhaustion score derived from baseline biopsy transcriptomics: those with barrier-dominant resistance could be randomized to SBRT plus ICI with or without anti-fibrotic agents, while those with exhaustion-dominant profiles would receive LDRT plus bispecific antibodies or ADCs. Co-primary endpoints could include intratumoral CD8^+^ T cell density (pharmacodynamic readout) and 6-month progression-free survival. This biomarker-driven approach, if validated, would represent a fundamental shift from empirical salvage therapy toward mechanism-guided precision radio-immunotherapy in esophageal cancer.
